# Prospective comparison of tadalafil 5 mg alone, silodosin 8 mg alone, and the combination of both in treatment of lower urinary tract symptoms related to benign prostatic hyperplasia

**DOI:** 10.1007/s00345-022-04071-7

**Published:** 2022-06-30

**Authors:** Mostafa AbdelRazek, Ahmad Abolyosr, Omar Mhammed, Atef Fathi, Mohammed Talaat, Ahmed Hassan

**Affiliations:** Urology Department, Qena University Hospital, South Valley University, Qena, P.O: 83523 Egypt

**Keywords:** Benign prostatic hyperplasia, Lower urinary tract symptoms of BPH, Tadalafil, Silodosin

## Abstract

**Background:**

Men with lower urinary tract symptoms (LUTS) associated with benign prostatic hyperplasia (BPH), will have deterioration in the quality of life. Likewise, BPH can be complicated by damage to bladder function, bladder stones formation, hematuria, and impaired kidney function. The goal of treatment is to avoid all those effects caused by BPH.

**Objective:**

To evaluate the efficacy of tadalafil alone, silodosin alone, and the combination of both in the treatment of LUTS associated with BPH.

**Patients and methods:**

Patients in our department with BPH who had LUTS were assigned randomly to three groups: **A** (101 patients) received tadalafil, 5 mg; **B** (102 patients) received silodosin, 8 mg; and group **C** (105 patients) received the combination of tadalafil, 5 mg, and silodosin, 8 mg. For all participants, we asses changes in the maximum urinary flow rate (Qmax), International Prostate Symptom Score (IPSS), International Index of Erectile Function (IIEF) score, Post-voiding urine (PVR) and all results were recorded and analyzed with the (SPSS) and Microsoft Excel 2010.

**Results:**

Qmax, IPSS, PVR and IIEF score improved significantly more with the combination of tadalafil and silodosin than with either drug alone (*p* < 0.001). Three months after treatment, the mean Qmax values were 14.4 ml/sec in group A, 15.2 ml/sec in group B, and 15.8 ml/sec in group C; and the mean IPSSs were 17.6 in group A, 16.7 in group B, and 15.6 in group C (*p* < 0.001).

**Conclusion:**

Tadalafil and silodosin are effective treatment options in men with BPH who have LUTS, but the combination of both is more effective and feasible in treating LUTS of BPH.

## Introduction

Benign prostatic hyperplasia (BPH) is the most common benign tumor in men. No convincing evidence of a positive correlation for any causal factors, other than age and the presence of testes, exists. Of men with microscopic and macroscopic evidence of BPH, 25–50% will develop clinical manifestations of BPH. The prevalence of clinical BPH in an individual community among men aged 55–74 years may vary from 5 to 30%. Results of some studies have suggested a genetic predisposition, whereas others have indicated racial differences [1].

According to the 2020 guidelines of the European Association of Urology, both α1 adrenoceptor blockers (α1-blockers) and phosphodiesterase type 5 inhibitors (PDE5-Is) are recommended as the first-line medical treatment for lower urinary tract symptoms (LUTS) associated with BPH [2]. Silodosin is a third-generation α1-blocker, and its effect on LUTS is more selective than that of other α1-blockers. However, in some men with BPH, silodosin treatment results in inadequate improvement in LUTS. Therefore, for such patients, we sought to identify further treatment strategies, including combination or add-on therapy with other drugs for LUTS [3].

Tadalafil is a PDE5-I used widely in the treatment of erectile dysfunction (ED) and was approved for the treatment of signs and symptoms of BPH (LUTS). The mechanisms of PDE5-I responsible for improvements in LUTS include the inhibition of PDE5 isoenzymes present in the bladder, prostate, urethra, and supporting vasculature, and consequent increases in the intracellular nitric oxide–cyclic guanosine monophosphate concentration, relaxation of the muscle cells in these structures, improved blood perfusion, and reduced afferent signaling from the urogenital tract [4].

Other medications, such as silodosin, improve short-term LUTS. Data are not yet available to assess their long-term efficacy or prevention of disease progression. Trials with longer durations of treatment and follow-up are needed to assess the effects of these therapies on response rates [5].

Objective of this study is to evaluate the efficacy of tadalafil alone, silodosin alone, and the combination of both in the treatment of LUTS associated with BPH.

## Patients and methods

**Fig. 1:**
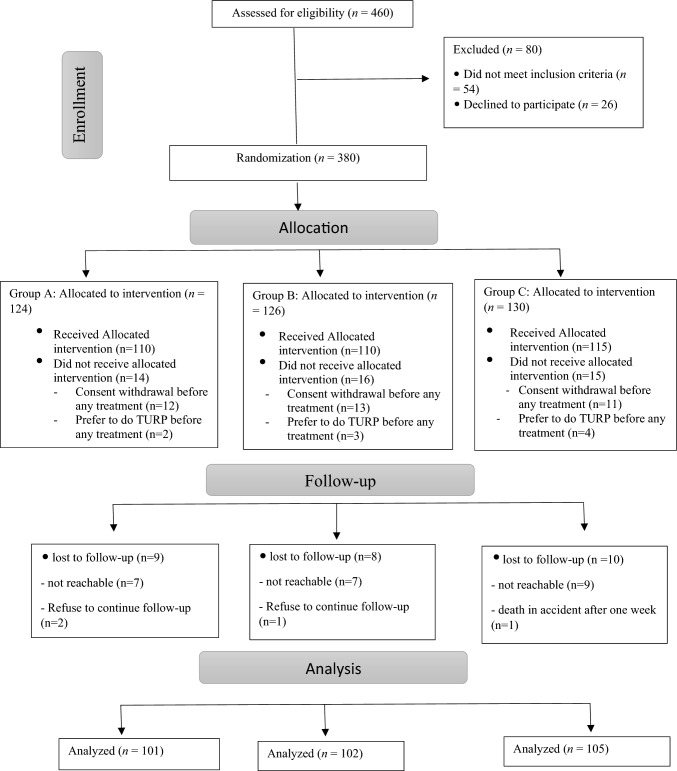
Consolidated Standard of Reporting Trials (CONSORT) diagram for patient assignment throughout the study

This was a prospective randomized study of patients who presented to our clinic with LUTS of BPH between December 2018 and December 2020. The patients were randomly (using block randomization method by Stata, version 13.1, StataCorp, for Microsoft Windows ®) allocated into three groups: patients in group A received tadalafil, 5 mg, once daily for 3 months; patients in group B received silodosin, 8 mg, once daily for 3 months; and patients in group C received both tadalafil, 5 mg, and silodosin, 8 mg, once daily, for 3 months (Fig. 1).

Inclusion criteria were age > 50 years; IPSS > 8; IIEF score > 12; Qmax < 10 mL/s and PVR urine < 200 cc. Exclusion criteria were the suspected presence of prostate cancer or bladder carcinoma in situ; complicated BPH (patients with refractory urinary retention, renal insufficiency, recurrent urinary tract infections, recurrent bladder stones or gross hematuria secondary to BPH); insufficient renal function (serum creatinine concentration > 2 mg/dL); cardiac, diabetics and patients with history of hypersensitivity to used drugs.

For all participants, we conducted history taking, including IPSS and IIEF scores; a complete physical examination, including digital rectal examination; laboratory investigations, including urinalysis and measurements of serum creatinine and serum prostate-specific antigen; abdominal ultrasonography to assess upper urinary tract prostate volume and presence of post-voiding residual (PVR) urine; uroflowmetry; and follow-up scheduling.

Patients in the two groups attended follow-up visits after 2 weeks and 1, 2, and 3 months of treatment. Repeated IPSS and IIEF scores were obtained. Uroflowmetry was performed to measure the maximum urinary flow rate Qmax, and abdominal ultrasonography was performed to assess PVR and prostate volume.

All results were recorded and analyzed using the Statistical Package for the Social Sciences® (SPSS; IBM version 22.0 Corporation, Armonk, NY, USA) and Microsoft Excel 2010 (Microsoft Corporation, Redmond, WA, USA).

Results were expressed as mean ± standard deviation or frequency (percentage) as appropriate. Mann–Whitney *U *test: was used when comparing between two means (for abnormal distributed data). Kruskal–Willis test (KW): when comparing between more than two means (for abnormally distributed data). Post hoc test least significant difference (LSD): was used for multiple comparisons between different variables. Probability (*P *value) *P *value < 0.05 was considered significant. *P *value < 0.001 was considered as highly significant. *P *value > 0.05 was considered insignificant.

## Results

In this study, we assess 460 patients for eligibility, after exclusions, group A consisted of 101 patients with LUTS of BPH who took tadalafil, 5 mg, for 3 months; their ages ranged from 52 to 75 years (mean, 61.7 ± 4.6 years). Group B consisted of 102 patients with LUTS of BPH who took silodosin, 8 mg, for 3 months; their ages ranged from 54 to 72 years (mean, 62.9 ± 5.6 years). Group C consisted of 105 patients with LUTS of BPH who took the combination of tadalafil, 5 mg, and silodosin, 8 mg, for 3 months; their ages ranged from 55 to 73 years (mean, 62.6 ± 5.4 years).

### Maximum urinary flow rate

Among the three groups, we found no significant difference (*p* = 0.851) in the mean Qmax before treatment (group A: 7.1 mL/sec; group B: 7.2 mL/sec; and group C: 7.1 mL/sec; Table 1). In each group, the Qmax differences before and 3 months after treatment were highly significantly different (*p* < 0.001).

**Table 1:** : Comparisons between studied groups as regards age, PSA, Pre-treatment (Qmax, IPSS, IIEF score)

Pre-treatment	Groups			KW	*P *value
	Group A (*n* = 101)	Group B (*n* = 102)	Group C (*n* = 105)		
Age (years)					
Mean	61.7	62.9	62.6	3.76	0.152
± SD	4.6	5.6	5.4		
PSA (ng/ml)					
Mean	2.3	2.2	1.9	2.7	0.254
± SD	1.4	1.4	1.2		
Prostate volume (g)					
Mean	65	57.5	60	2.67	0.192
± SD	15.3	17.8	16.2		
Qmax					
Mean	7.1	7.2	7.1	0.322	0.851
± SD	1.2	1.2	1.2		
IPSS					
Mean	21.2	20.8	21.3	2.37	0.304
± SD	1.8	2.0	1.8		
IIEF score					
Mean	15.0	14.5	14.9	3.07	0.215
± SD	2.0	1.7	2.0		
PVR					
Mean	50.8	51.0	51.0	0.094	0.954
± SD	6.1	5.9	5.7		

Three months after treatment, the mean Qmax values in the three groups—14.4 ml/sec in group A, 15.2 ml/sec in group B, and 15.8 ml/sec in group C—were significantly different (each *p* < 0.001). Post hoc tests revealed highly significant differences between groups A and B and between groups A and C, as well as a significant difference between groups B and C. Group C exhibited the best improvement in the Qmax, followed by groups B and A (Table 2). We also found highly significant differences (all *p*’s < 0.05) between pretreatment and posttreatment difference ΔQmax; post hoc tests revealed a significant difference (*p* = 0.002) between groups A and B, a highly significant difference (*p* < 0.001) between groups A and C, and no significant difference (*p* = 0.069) between groups B and C (Table 3).

**Table 2:** comparisons between studied groups as regards ∆ Q Max, ∆ IPSS, ∆ IIEF score, ∆ PVR

	Groups			KW	*P *value
	Group A (*n* = 101)	Group B (*n* = 102)	Group C (*n* = 105)		
∆ Q Max					
Mean	7.2	8.1	8.7	24.04	< 0.001
± SD	2.0	2.3	2.0		
∆ IPSS					
Mean	3.6	4.1	5.6	30.9	< 0.001
± SD	1.9	2.2	2.8		
∆ IIEF score					
Mean	5.9	7.4	7.0	26.8	< 0.001
± SD	2.4	2.1	2.3		
∆ PVR					
Mean	11.1	13.3	15.5	24.6	< 0.001
± SD	7.1	6.4	6.4

**Table 3:** (A) Post hoc test for multiple comparisons between studied groups as regard Q Max, IPSS, IIEF, PVR. (3 months after treatment). (B) Post hoc test as regards ∆ Q Max, ∆ IPSS, ∆ IIEF, ∆ PVR

	Qmax		IPSS		IIEF score		PVR	
Groups	LSD (least significance difference)	*P *value	LSD	*P *value	LSD	*P *value	LSD	*P *value
Group A								
Group B	−0.88	< 0.001	0.85	0.003	−0.65	0.002	2.22	< 0.001
Group C	−1.45	< 0.001	1.92	< 0.001	−1.11	< 0.001	4.10	< 0.001
Group B								
Group A	0.88	< 0.001	−0.85	0.003	0.65	0.002	−2.22	< 0.001
Group C	−0.57	0.01	1.07	< 0.001	−0.46	0.016	1.88	0.001
Group C								
Group A	1.45	< 0.001	−1.92	< 0.001	1.11	< 0.001	−4.10	< 0.001
Group B	0.57	0.01	−1.07	< 0.001	0.46	0.016	−1.88	0.001

	∆ Qmax		∆ IPSS		∆ IIEF		∆ PVR	
Groups	LSD (least significance difference)	*P* value	LSD	*P *value	LSD	*P *value	LSD	*P *value
Group A								
Group B	0.91	0.002	−0.46	0.157	−1.5	< 0.001	−2.16	0.02
Group C	1.45	< 0.001	−1.99	< 0.001	−1.14	< 0.001	−4.4	< 0.001
Group B								
Group A	−0.91	0.002	0.46	0.157	1.5	< 0.001	2.16	0.02
Group C	0.53	0.069	−1.5	< 0.001	0.39	0.215	−2.2	0.014
Group C								
Group A	−1.45	< 0.001	1.99	< 0.001	1.14	< 0.001	4.4	< 0.001
Group B	−0.53	0.069	1.5	< 0.001	−0.39	0.215	2.2	0.014

### International prostate symptom score

We found no significant difference in the IPSS (*p* 0.304) before treatment among the three groups. The mean IPSSs were 21.2 in group A, 20.8 in group B, and 21.3 in group C (Table 1). In each group, the IPSSs before and 3 months after treatment were significantly different (*p* < 0.001).

Three months after treatment, the mean IPSSs were highly significantly different (*p* < 0.001) among the three groups: 17.6 in group A, 16.7 in group B, and 15.6 in group C (Table 2). Post hoc tests revealed a significant difference between groups A and B (*p* 0.003) and highly significant differences between groups A and C (*p* < 0.001) and between groups B and C (*p* < 0.001). Group C exhibited the best improvement in the IPSS, followed by groups B and A). We also found a highly significant difference (*p* < 0.05) between pretreatment and posttreatment difference Δ IPSSs; post hoc tests revealed no significant difference (*p* 0.157) between groups A and B, but highly statistically significant differences (both *p*’s < 0.001) between groups A and C and between groups B and C (Table 3).

### International index of erectile function score (IIEF)

We found no significant difference (*p* 0.215) in the mean IIEF score before treatment among all three groups. The mean IIEF scores were 15 in group A, 14.5 in group B, and 14.9 in group C (Table 1). In each group, IIEF scores before and 3 months after treatment were significantly different (*p* < 0.001).

Three months after treatment, the mean IIEF scores were highly significantly different.

(*p* < 0.001) among the three groups: 20.8 in group A, 21.5 in group B, and 21.9 in group C (Table 2). Post hoc tests revealed a significant difference between groups A and B (*p *0.002), a highly significant difference between groups A and C (*p* < 0.001), and a significant difference between groups B and C (*p* < 0.016). Group C exhibited the best improvement in IIEF scores, followed by groups A and B.

We also found a highly significant difference (*p* < 0.05) between pretreatment and posttreatment difference **Δ**IIEF scores; post hoc tests revealed highly significant differences (both *p*’s < 0.001) between groups A and B and between groups A and C, but no significant difference (*p* 0.215) between groups B and C (Table 3).

### Post-voiding residual urine

We found no significant difference in the mean pretreatment PVR urine measurements among all three groups (Table 1). In each group, however, PVR urine measurements before and after treatment were highly significantly different (all *p*’s < 0.001). The differences between pretreatment and posttreatment PVR urine measurements were highly significantly different in all three groups (all *p*’s < 0.05). Post hoc tests revealed a statistically significant difference (*p* < 0.02) between groups A and B, a highly statistically significant difference (*p* < 0.001) between groups A and C, and a statistically significant difference (*p* 0.014) between groups B and C (Table 2). Group C exhibited the best improvement in PVR urine measurements, followed by groups B and A.

### Complications

We found no statistically significant difference between the study groups with regard to complications, except for retrograde ejaculation. Retrograde ejaculation occurred among only 9.5% of patients receiving combination therapy, compared with 5.9% of patients receiving silodosin alone (Table 4). There was no incidence of acute retention or prostatitis during treatment period.

**Table 4:** Comparisons between studied groups regarding complications

	Groups							*P* value
	Group A (*n* = 101) %		Group B (*n* = 102) %		Group C (*n* = 105) %			
Retrograde ejaculation	0	0	6	5.9	10	9.5	9.6	0.151
Headache	4	4	5	4.9	5	4.8	0.12	0.396
Nasal congestion	2	2	3	2.9	3	2.9	0.22	0.892
Ocular hyperemia	2	2	3	2.9	3	2.9	0.22	0.892
Myalgia	2	2	3	2.9	3	2.9	0.22	0.892
Dizziness	1	1	2	2	2	1.9	0.37	0.941
Palpitation	0	0	1	1	2	1.9	1.93	0.892
Dyspepsia	0	0	2	2	2	1.9	1.97	0.892
Peripheral edema	0	0	1	1	1	1	0.98	0.884
Flushing	3	3	2	2	2	1.9	0.32	0.827
Orthostatic hypotension	0	0	1	1	1	1	0.98	0.380
TTT discontinuation due to AEs	0	0	2	2	4	3.8	3.9	0.371

## Discussion

In this study, we found that the daily administration of tadalafil, 5 mg, was effective in improving the overall urinary symptoms, but improvement was better when tadalafil was combined with silodosin, 8 mg. This was also true for uroflowmetry parameters, such as the Qmax, PVR and voiding IPSS.

In elderly men, ED and LUTS related to benign prostatic enlargement are highly prevalent, have adverse effects on patients’ QOL, and impose a significant economic burden. Preclinical and clinical trials have demonstrated that besides aging, several metabolic factors affect the onset and worsening of both ED and LUTS, contributing to penile and nerve alterations and prostate enlargement and inflammation. Although the pathophysiological pathways affected in both ED and LUTS are still not totally elucidated, PDE5-Is have proved to be effective for the treatment of both these conditions [6]. In fact, PDE5-Is can manage prostate inflammation and may ameliorate the related fibrosis by improving pelvic and prostate oxygenation. Moreover, they might help restore the physiological activity of the prostate and help stabilize the glandular structural anatomy [7].

Several randomized controlled trials have demonstrated that PDE5-Is can significantly decrease the IPSS score, ameliorate both storage and voiding LUTS, and improve patients’ QOL. However, in most trials, their effects on the Qmax did not differ significantly from those on the placebo. Likewise, according to a meta-analysis by Gacci et al. (2012), IPSS and IIEF scores, but not Qmax, were improved significantly by PDE5-Is. In the first systematic review of the use of PDE5-Is for LUTS associated with benign prostatic enlargement, Laydner et al. reported that PDE5-Is improve IPSS and IIEF-5 scores but not Qmax [8]. However, in a study by Roehrborn et al. (2014), who evaluated the efficacy of tadalafil, 5 mg, once daily, a slight but significant Qmax increase was observed [9].

Moreover, in a subset analysis based on data from a systematic review, the daily administration of tadalafil, 5 mg, was associated with remarkable improvements in both ED and LUTS of BPH. Thus, according to the European Association of Urology guidelines, tadalafil, 5 mg, is currently considered another valuable treatment option for men with moderate to severe LUTS suggestive of BPH [2]. In clinical practice, tadalafil is increasingly prescribed as the first-line therapy for LUTS of BPH and concomitant ED. However, patients with prevalent voiding LUTS often switch to other medical treatments; ongoing tadalafil/silodosin combination therapy is rarely considered, although combination therapy has been proposed by several authors [10].

Changes in the erectile function after treatment with silodosin 8 for 3 months (group B) revealed different results with those previously reported in the literature [11, 12] as these clinical studies demonstrated either impairment or no change with Silodosin 8 mg per day in the overall satisfaction domains of the IIEF at 12 weeks [11, 12]. However, we obtained improved results of the patients at evaluation of the third month which was consistent with Cihan et al. [13]; we attribute this improvement to that the worse effect of LUTS on QOL and sexual life and also these results explained by Bastaskin et al. [14] experimental study showing improvements in the erectile functions of partially obstructed rats in their bladder outlet through Silodosin administration and also demonstrated that the neuronal nitric oxide synthase pathway has mediated the effects of Silodosin on cavernosal recovery. Another randomized controlled trial by Sertkaya et al., reported the nocebo effect occurs in patients informed of the potential side effects of silodosin, with frequently reported ED which supports that improvement of QOL and its psychological impact may have role in improvement results after Silodosin use [15]. Therefore, we conclude that larger clinical trials may support our findings of improvement of the erectile function with Silodosin treatment.

We found that both subjective and objective parameters were improved significantly at the end of the trial in the three treatment arms, which supported the use of tadalafil, 5 mg, as monotherapy or in combination with silodosin, 8 mg, in men with ED and LUTS. However, at the end of the trial, the Qmax, PVR and voiding IPSS were significantly better in men treated with combination therapy than in those treated with tadalafil alone.

Tadalafil alone significantly decreased the total IPSS and improved Qmax after 12 weeks; nevertheless, improvements in voiding symptoms were better with the combination of tadalafil and silodosin. The first meta-analysis of studies of PDE5-Is for the treatment of ED and LUTS proved the improvements in the IPSS score and Qmax, in addition to the IIEF score, in men treated with the association of alpha blockers and PDE5-Is compared with those treated with alpha blockers alone [16].

The results of our study suggest that the combination of silodosin and tadalafil helped achieve further improvements in the Qmax, PVR and voiding symptoms, compared with tadalafil monotherapy, after 12 weeks. However, as the overall improvements in LUTS IPSS and ED (IIEF) were similar in the three treatment arms, therapy may become increasingly patient-oriented and personalized.

Similar findings were shown by Kaplan et al., in a randomized trial in which they compared alfuzosin, sildenafil, and their combination. After 12 weeks of treatment, the rates of improvement in Qmax were 21% for combination therapy, 6% for sildenafil, and 11% for alfuzosin [17].

Our results, in agreement with the previous studies, proved that voiding LUTS and uroflowmetric parameters could be improved significantly with the addition of alpha blockers to PDE5-I therapy. Thus, depending on the prevailing LUTS reported at the first or follow-up visit, more precise counseling and medications might be offered in daily clinical practice. Tadalafil, 5 mg daily, was well tolerated both in monotherapy and in combination with silodosin, 8 mg.

### Limitations of the study

The number of patients was not large enough to reduce the effect of statistical error during analysis. Also, for the short follow-up period (12 weeks), however, it is not affecting our results accuracy as maximum drugs effect achieved within 6–8 weeks of treatment.

Patients’ comorbidities should have been considered, but patients with major comorbidities affecting LUTS/BPH were excluded from the study. Another limitation of this study is not placebo-controlled study. More clinical trials to compare the association of tadalafil and other pharmacological agents used in LUTS of BPH, such as 5-alpha-reductase inhibitors, β3-adrenoceptor agonists, and muscarinic receptor antagonists, are needed so that the therapeutic strategies for patients with LUTS of BPH can be personalized.

## Conclusion

Either tadalafil, 5 mg daily, or silodosin, 8 mg daily, improves overall LUTS after 12 weeks. However, the combination of both is effective in improving voiding symptoms and the Qmax. Although the overall occurrence of side effects is slightly higher with the combination than with tadalafil alone, their low severity enables good compliance and implies safety; hence, we recommend combination therapy, especially tadalafil with silodosin, in patients with LUTS of BPH, with or without ED.

### Recommendations

1. Increase the number of patients in each group.

2. Prolong the follow-up period of patients.

3. Use combination therapy in patients with LUTS of BPH, especially those with ED.
