# Correction: Prospective comparison of tadalafil 5 mg alone, silodosin 8 mg alone, and the combination of both in treatment of lower urinary tract symptoms related to benign prostatic hyperplasia

**DOI:** 10.1007/s00345-024-05164-1

**Published:** 2024-07-22

**Authors:** Mostafa AbdelRazek, Ahmad Abolyosr, Omar Mhammed, Atef Fathi, Mohammed Talaat, Ahmed Hassan

**Affiliations:** Urology Department, Qena University Hospital, South Valley University, Qena, P.O: 83523 Egypt

**Correction: World Journal of Urology (2022) 40:2063–2070** 10.1007/s00345-022-04071-7

In the original publication of the article, Group B and Group A in Row Group C was interchanged, and published incorrectly in Table 3. The correct Table 3 is given below:

**Table 3:** (A) Post hoc test for multiple comparisons between studied groups as regard Q Max, IPSS, IIEF, PVR. (3 months after treatment). (B) Post hoc test as regard ∆ Q Max, ∆ IPSS, ∆ IIEF, ∆ PVR

Groups		Q Max		IPSS		IIEF score		PVR	
		LSD (least significance difference)	*p* value	LSD	*p* value	LSD	*p* value	LSD	*p* value
Group A	Group B	− 0.88	< 0.001	0.85	0.003	− 0.65	0.002	2.22	< 0.001
	Group C	− 1.45	< 0.001	1.92	< 0.001	− 1.11	< 0.001	4.10	< 0.001
Group B	Group A	0.88	< 0.001	− 0.85	0.003	0.65	0.002	− 2.22	< 0.001
	Group C	− 0.57	0.01	1.07	< 0.001	− 0.46	0.016	1.88	0.001
Group C	Group A	1.45	< 0.001	− 1.92	< 0.001	1.11	< 0.001	− 4.10	< 0.001
	Group B	0.57	0.01	− 1.07	< 0.001	0.46	0.016	− 1.88	0.001

Groups		∆ Q Max		∆ IPSS		∆ IIEF		∆ PVR	
		LSD (least significance difference)	*p* value	LSD	*p* value	LSD	*p* value	LSD	*p* value
Group A	Group B	0.91	0.002	− 0.46	0.157	− 1.5	< 0.001	− 2.16	0.02
	Group C	1.45	< 0.001	− 1.99	< 0.001	− 1.14	< 0.001	− 4.4	< 0.001
Group B	Group A	− 0.91	0.002	0.46	0.157	1.5	< 0.001	2.16	0.02
	Group C	0.53	0.069	− 1.5	< 0.001	0.39	0.215	− 2.2	0.014
Group C	Group A	− 1.45	< 0.001	1.99	< 0.001	1.14	< 0.001	4.4	< 0.001
	Group B	− 0.53	0.069	1.5	< 0.001	− 0.39	0.215	2.2	0.014

The percent numbers between sildosen group and combination group in the text of Complications section were interchanged, and published incorrectly. The correct data is given below:

**Complications**

We found no statistically significant difference between the study groups with regard to complications, except for retrograde ejaculation. Retrograde ejaculation occurred among only 9.5% of patients receiving combination therapy, compared with 5.9% of patients receiving silodosin alone (Table 4). There was no incidence of acute retention or prostatitis during treatment period.

The original article has been corrected accordingly.

